# A Novel Variant in *ABCD1* Gene Presenting as Adolescent-Onset Atypical Adrenomyeloneuropathy With Spastic Ataxia

**DOI:** 10.3389/fneur.2018.00271

**Published:** 2018-04-23

**Authors:** Yanxing Chen, Jianfang Zhang, Jianwen Wang, Kang Wang

**Affiliations:** ^1^Department of Neurology, The Second Affiliated Hospital, School of Medicine, Zhejiang University, Hangzhou, China; ^2^Department of Neurology, The First Affiliated Hospital, School of Medicine, Zhejiang University, Hangzhou, China

**Keywords:** X-linked adrenoleukodystrophy, adrenomyeloneuropathy, spastic ataxia, atypical, *de novo*

## Abstract

X-linked adrenoleukodystrophy (X-ALD) is a rare neurological disorder with a highly complex clinical presentation. Adrenal function, spinal cord, peripheral nerves, and cerebral white matter are commonly affected in adult-onset male patients. Here, we report a family with unusual presentation of X-ALD. The 19-year-old proband had presented with atypical symptoms of adrenomyeloneuropathy (AMN) for 3 years, only with spastic paraparesis, cerebellar ataxia, and cerebellar atrophy with white matter hyperintensity. It is rare for an AMN male patient to present the initial symptoms at such an early age with the adrenal function, sphincter function, and dorsal column of the spinal cord spared. He is also the youngest male AMN patient reported to have cerebellar ataxia. His mother also presented unusually early onset of the similar manifestations. A novel variant c.1144A>C (p.Thr382Pro) in exon 3 of the *ABCD1* gene was identified. Family study involving the grandparents of the proband revealed the *de novo* occurrence of the variant in the mother.

## Introduction

X-linked adrenoleukodystrophy (ALD) is a peroxisomal disorder which is characterized by accumulation of saturated very long chain fatty acids (VLCFA, ≥C22:0) in plasma and all tissues, including the white matter of the brain, the spinal cord, and adrenal cortex (1). It is caused by the absence or dysfunction of the ATP-binding cassette sub-family D member 1 (also known as ALDP), which results from variations in the *ABCD1* gene located on the X-chromosome (2). ALDP deficiency leads to impaired peroxisomal β-oxidation of VLCFAs, and consequently its accumulation in different tissues (1). The clinical phenotypes of X-ALD vary considerably among individual patients (3). Rapid progressive and devastating cerebral demyelination in childhood and slowly progressive myelopathy in adulthood (also known as adrenomyeloneuropathy, AMN) are the two most commonly seen phenotypes. Cerebral ALD also occurs in adolescence or adulthood, but less frequently. Most (>80%) heterozygous female carriers of ALD will develop signs and symptoms of myelopathy and/or peripheral neuropathy by the age of 60 years (4).

Here, we report an X-ALD family with the two affected family members presenting with spastic ataxia. Brain magnetic resonance imaging (MRI) revealed significant cerebellar atrophy. A novel variant c.1144A>C (p.Thr382Pro) in exon 3 of the *ABCD1* gene of the proband, which was derived from a *de novo* variation in his mother, was identified.

## Case Report

**Figure 1:**
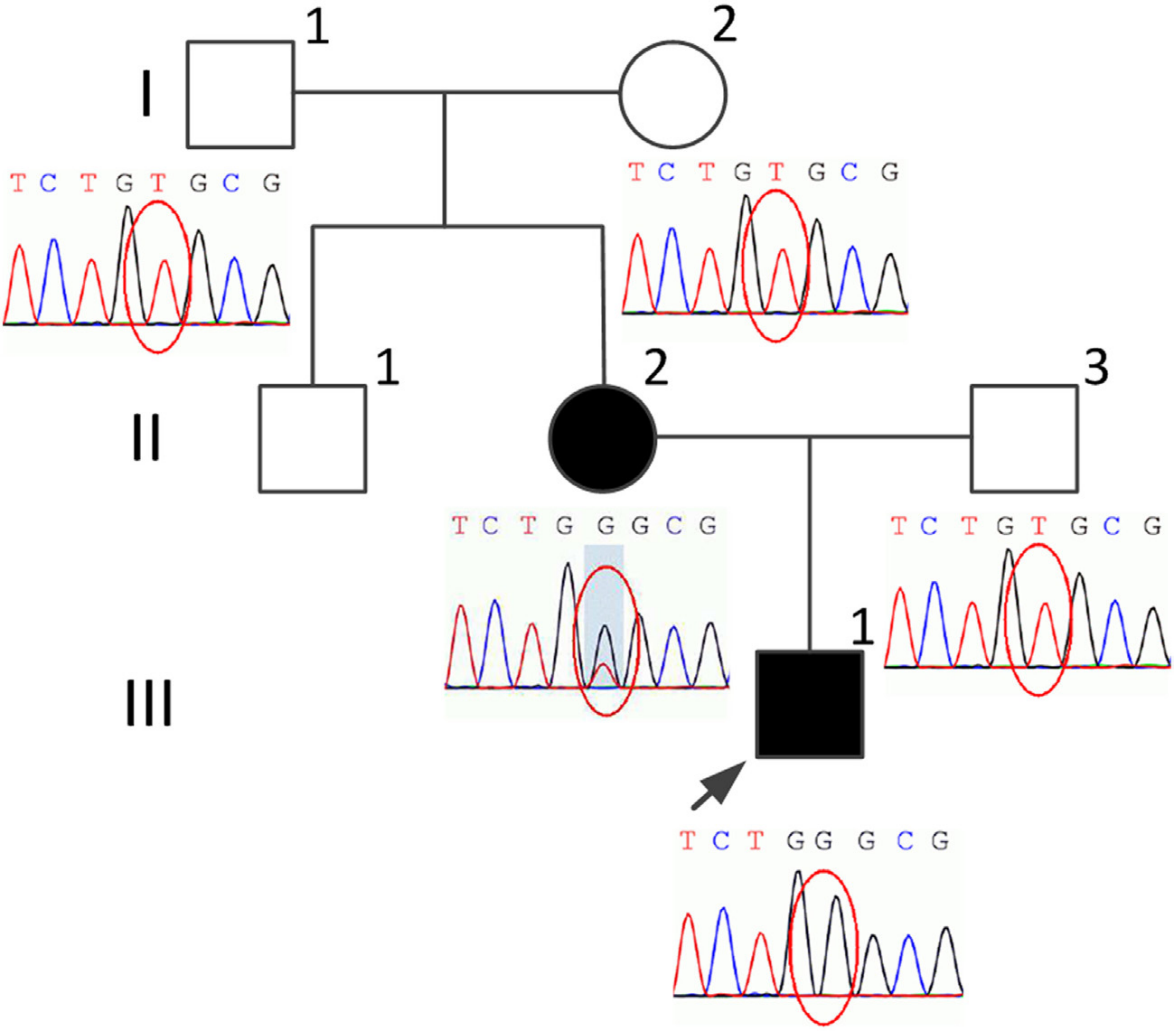
Pedigree of the present family and the variant screening diagrams. The proband is indicated by an arrow. Black indicates affected. Squares indicate males. Circles indicate females.

The proband (Figure 1, III-1), a 19-year-old male college student presented with a history of slowly progressive gait disorder for 3 years. On neurological examination, spasticity and hyperactive tendon reflexes with Hoffman and Babinski sign in the limbs were observed. The patient also presented dysmetria on finger-to-nose test. He was unable to perform heel-to-shin test due to spasticity of the lower limbs, but can still walk unassisted. He was alert without any intellectual disabilities. His speech was fluent and clear without detectable dysarthria. His hearing and vision were normal. There was no nystagmus or ophthalmoplegia. Sensation, including the superficial and proprioception, was intact. General physical examination revealed brachydactyly on the left fourth toe. No skin rash or pigmentation was observed. The sphincter function was normal.

**Figure 2:**
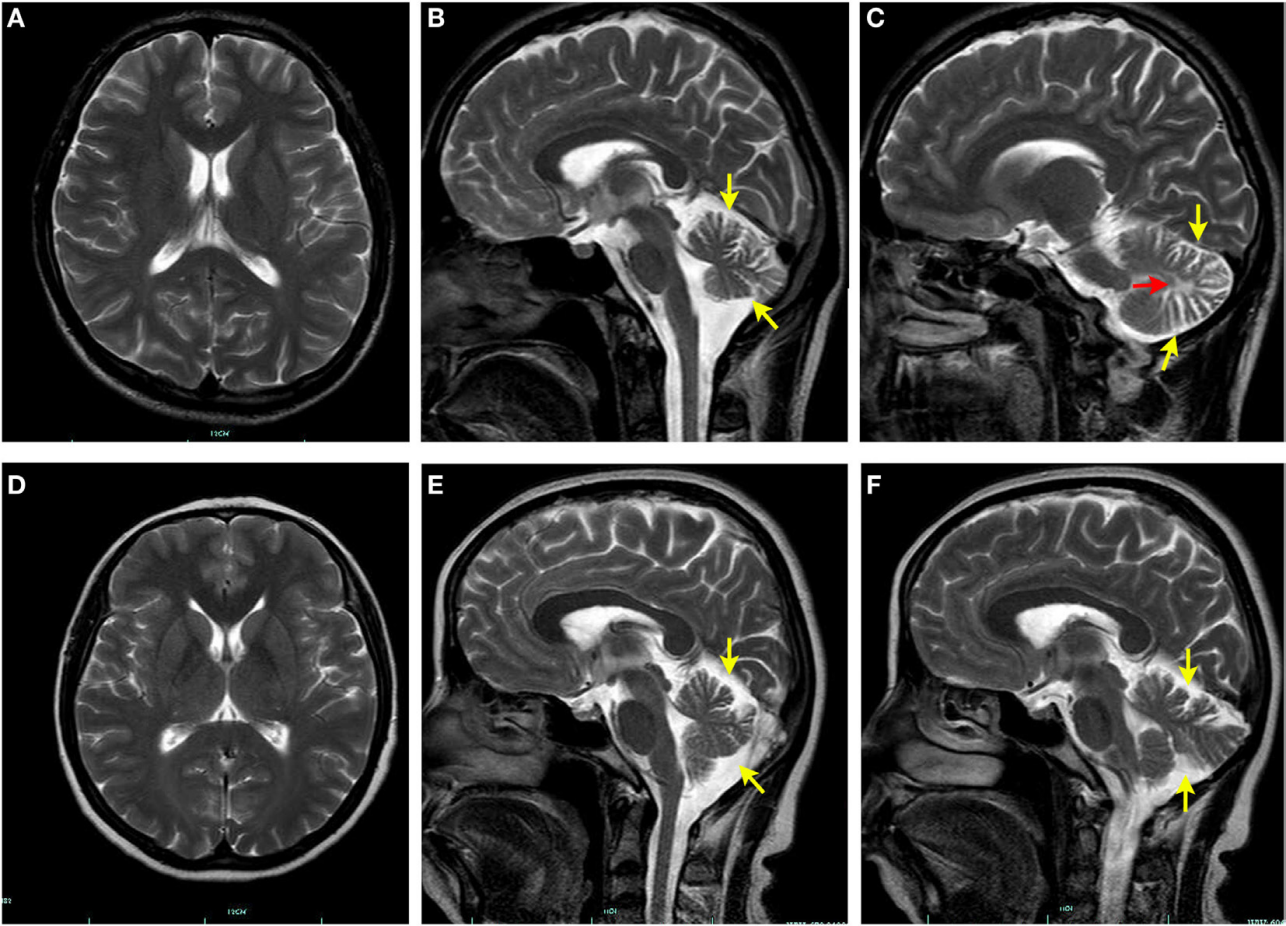
Brain magnetic resonance imaging (MRI) findings. No signal changes are seen in the axial T2-weighed brain MRI of the proband **(A)** and his mother **(D)**. Sagittal T2-weighed brain MRI of the proband **(B,C)** and his mother **(E,F)** shows manifest cerebellar atrophy (yellow arrow). White matter hyperintensity (red arrow) is also evident in cerebellum of the proband **(C)**.

Laboratory investigations, including full blood counts, blood electrolytes, erythrocyte sedimentation rate, thyroid function, sex hormone, liver and renal function, and pituitary hormone levels were normal. The plasma adrenocorticotropic hormone and cortisol levels were also within the normal range. MRI revealed obvious cerebellar atrophy and mild bilateral cerebellar white matter signal changes (Figure 2). MRI of the spinal cord did not show any atrophy, signal changes, or compression. Electrophysiological studies, including nerve conduction, visual evoked potentials, brainstem auditory evoked potentials, and somatosensory evoked potentials, did not reveal any abnormal findings. The plasma VLCFA assay revealed the following results: C22:0 43.59 μmol/L (normal range 32.0 ± 9.39 μmol/L), C24:0 74.13 μmol/L (normal range 27.5 ± 9.17 μmol/L), C26:0 2.611 μmol/L (normal range 0.51 ± 0.132 μmol/L), C24:0/C22:0 ratio 1.7 (normal range 0.883 ± 0.277), and C26:0/C22:0 ratio 0.06 (normal range 0.017 ± 0.006), suggesting elevated plasma VLCFAs. Based on these findings a diagnosis of X-ALD was made.

His 45-year-old mother (Figure 1, II-2) had the similar manifestations, which initially occurred at the age of 25 years. She needed to use the wheelchair 3 years ago, but could still ambulate with the aid of crutches. However, she only had some signs and symptoms of myelopathy with very mild cerebellar ataxia. The adrenal function, sphincter function, peripheral nerves, dorsal column, and cerebral white matter were spared, without any detectable disturbances (Figure 2). An increased plasma VLCFA profile with the ratios of C24:0/C22:0 of 1.5 and C26:0/C22:0 of 0.085 was also observed. His grandparents (I-1 and I-2), uncle (II-1), and father (II-3) (Figure 1) do not have any clinical abnormalities or increased plasma VLCFA.

Genomic DNA was isolated from peripheral blood of the family members. Target next-generation sequencing was performed, followed by Sanger sequencing. We found a novel variant c.1144A>C (p.Thr382Pro) in exon 3 in the *ABCD1* gene in the proband (III-1) and his mother (II-2). The mother (II-2) was heterogygous for this variant. His uncle (II-1) declined DNA analysis. This variant was defined as variant of uncertain significance according to the American College of Medical Genetics and Genomics guidelines. Polymorphism phenotyping (PolyPhen-2^1^) using sequence- and structure-based information to predict the effect of variants was also applied. This variant was predicted to be probably damaging with a score of 0.996.

## Discussion

We report here that a young adult male and his mother both presented spastic paraparesis and mild cerebellar ataxia, and cerebellar atrophy on MRI. Their manifestations were very alike. The age of onset of the initial symptoms were around 20 years, and were limited to the spinal cord and cerebellum. The diagnosis of X-ALD was made based on the clinical features and high levels of plasma VLCFAs.

The incidence rate of X-ALD is between 1:20,000 and 1:30,000, without major differences among countries across the world (5). The rarity of the disease is one of the reasons why many adulthood-onset patients are misdiagnosed as other diseases like hereditary spastic paraplegia and multiple sclerosis (MS), especially for symptomatic heterozygous female carriers. The clinical phenotypes vary greatly among male patients, and can be classified into the following categories: addison-only, cerebral ALD, AMN, and asymptomatic form (3). These phenotypes are not static, but can evolve over time. Addison-only males most likely will progress to develop AMN and/or cerebral ALD. Virtually all patients with X-ALD who reach adulthood develop AMN, usually in the third and fourth decades of life (3, 6). It is rare for an adolescent to have AMN as the initial manifestation. Approximately 20% of AMN patients may develop cerebral ALD (7), which is rapid progressive and devastating. AMN is the most common form of X-ALD in adulthood. Patients with AMN usually develop progressive spastic paraparesis, dorsal column dysfunction, peripheral neuropathy, sphincter disturbance, and adrenal insufficiency. The dorsal column dysfunction is usually prominent. The sphincter disturbance, mostly urinary dysfunction, initially presenting as urge complaints, progressing to full incontinence, is almost invariably present (8). The adrenal insufficiency was even identified in 86% of asymptomatic boys in a prospective study (9). Therefore, as for the proband, given the age of onset at 16, the absence of other symptoms of AMN, and the lack of adrenal insufficiency, his manifestation was atypical and can easily be misdiagnosed. The cerebellum involvement, which is rare, has been reported predominantly in Asian X-ALD patients (6), accounting for 0.39% of adult-onset cerebellar ataxia (10). The awareness of ataxia variant in Asian X-ALD patients makes it necessary to have X-ALD as a differential diagnosis in patients suspected with the cerebellar subtype of multiple system atrophy or spinocerebellar ataxia (SCA). However, the age of onset reported for cerebellum involvement in X-ALD ranged from 20 to 54 years (11). In the current study, the 19-year-old proband, manifested with a significant atrophy of the cerebellum on MRI, had already presented with a history of gait disturbance for 3 years. To the best of our knowledge, this is the first X-ALD case with cerebellum involvement at such an early age. It is not certain if the clinical manifestation of proband will progress to involve the adrenal gland and other areas of the nervous system. Follow-up on a regular basis is needed to recognize future progression of the disease to provide prompt symptomatic treatment, though disease modifying therapies are currently not available.

Unlike many other X-linked diseases, the female carriers of which remain asymptomatic, 88% heterozygous X-ALD women carriers show some degree of neurologic involvement by the age of >60 years (4). The symptoms are very similar to those of adult males with AMN. The onset of symptoms commonly occur around 50 years old (4, 12). It is very rare for women to be symptomatic at 25 years old. Besides, to the best of our knowledge, cerebellum involvement has only been reported in male patients (6, 10), this is the first study reporting a symptomatic female carrier with cerebellar ataxia. Even 20 years after the initial symptoms occur, the mother we report here can still walk with the help of the crutches. Besides, the phenotype did not evolve to develop other symptoms apart from spastic paraparesis and mild cerebellar ataxia. It has been speculated that skewed X-inactivation in females may account for the disease course and severity of the symptoms. However, as for X-ALD, the role of skewed X-inactivation is still controversial. More recent studies failed to demonstrate the association of variation severity and inactivation patterns with neurologic status (4, 12).

The plasma VLCFA level is a very important diagnostic biomarker for X-ALD. It is elevated in nearly all the male patients regardless of the presence or absence of clinical symptoms, and also in 85% female carriers (13). VLCFA levels are increased at the day of birth, and do not vary with age, providing the opportunity for mass newborn screening. No correlation exists between VLCFA levels and X-ALD phenotypes (4, 13). Besides, X-ALD is also characterized by the absence of genotype–phenotype correlation. Patients carrying the identical *ABCD1* gene variants could have markedly divergent clinical phenotypes (5, 8). Therefore, it is suggested that environmental triggers together with other modifier genes may contribute to the phenotypic variability of X-ALD (5, 8).

So far, more than 750 different variants in the *ABCD1* gene have been identified, as listed in the X-ALD database.^2^ DNA analysis revealed a novel variant c.1144A>C (p.Thr382Pro) in exon 3 of the *ABCD1* gene in the proband in the present study. His mother was heterozygous for the same variant, while the rest of the family did not have this variant, indicating *de novo* occurrence of the variant in the mother. Besides, the manifestations of female X-ALD patients are usually mild and not specific, without the involvement of the adrenal insufficiency and cerebral white matter demyelination. And it should be noted that in at least 10–15% of the females with ALD, VLCFA levels are within normal limits (13). Therefore, it is of particular difficulty for a symptomatic female carrier without a family history to be promptly diagnosed. In the current case, the mother was not diagnosed until the son became symptomatic. It is reported that a considerable amount of cases are with *de novo* variants, the rate of which varies from 5 to 19% (5). Therefore, it is recommended to consider X-ALD as a differential diagnosis for a female patient with mild paraparesis. Because her daughters or sons have a 50% risk of being carriers or being affected, early recognition will be important for management and genetic counseling.

The treatments for the majority of X-ALD patients mainly focus on symptomatic therapy. Hematopoietic stem cell transplantation (HSCT) has been used as a treatment for children with cerebral ALD, but should be performed at an early stage of cerebral disease. However, HSCT does not seem to prevent the onset of myelopathy and peripheral neuropathy in adulthood (14). Trials on adult cerebral X-ALD patients have only begun recently. It seems that HSCT can arrest the progression of cerebral demyelination and improve the neurological symptoms, but with a relative high transplant-related mortality (15). Currently, there is no indication for the use of HSCT in men with AMN or women with X-ALD. Lorenzo’s oil coupled with low-fat diet can lower VLCFA levels, but failed to improve neurological symptoms in X-ALD patients or arrest the progression of the disease (8). One study demonstrated a reduced risk of developing cerebral ALD in boys treated with Lorenzo’s oil (8, 16). Therefore, Lorenzo’s oil is offered to boys with ALD in some countries.

In conclusion, we report a family with an unusual manifestation of X-ALD. The proband presented with adolescent-onset spastic ataxia, and cerebellar atrophy with white matter hyperintensity. The mother, a heterozygous carrier, also presented unusually early onset of the similar manifestation. A novel variant in *ABCD1* gene was identified, which is a *de novo* variation in the mother. It is important to consider X-ALD as a differential diagnosis for patients with slowly progressive spastic ataxia.

## Data Availability Statement

The raw data supporting the conclusions of this manuscript will be made available by the authors, without undue reservation, to any qualified researcher.

## Ethics Statement

Written informed consent to publish the report was obtained from each member of the family.

## Author Contributions

YC, JZ, JW, and KW have actively participated in the data acquisition and interpretation, and they all commented and approved the final version of the manuscript. YC analyzed the data and drafted the manuscript. KW designed the study and revised the manuscript.

## Conflict of Interest Statement

The authors declare that the research was conducted in the absence of any commercial or financial relationships that could be construed as a potential conflict of interest.

## Footnotes

^1^http://genetics.bwh.harvard.edu/pph2/ (Accessed: February 13, 2018).

^2^http://www.x-ald.nl (Accessed: February 13, 2018).
